# Towards an indigenous definition of health: an explorative study to understand the indigenous Ecuadorian people’s health and illness concepts

**DOI:** 10.1186/s12939-020-1142-8

**Published:** 2020-06-22

**Authors:** Estefanía Bautista-Valarezo, Víctor Duque, Adriana Elizabeth Verdugo Sánchez, Viviana Dávalos-Batallas, Nele R. M. Michels, Kristin Hendrickx, Veronique Verhoeven

**Affiliations:** 1 Departamento de Ciencias de la Salud, Universidad Técnica Particular de Loja, San Cayetano alto s/n, 1101608 Loja, CP Ecuador; 2 Department of Primary and Interdisciplinary Care, Faculty of Medicine and Health Sciences, University of Antwerp, Universiteitsplein 1, Antwerp, Belgium; 3 Universidad de Cuenca, Cuenca, Ecuador

**Keywords:** Latin America, Traditional medicine, Indigenous, Health, Illness

## Abstract

**Backgrounds:**

An intercultural society facilitates equitable and respectful interrelations. Knowing and understanding each other’s sociocultural and linguitic contexts is a prerequisite for an intercultural society. This study explores the concepts of health and illness among healers of indigenous ethnicities in Southern Ecuador.

**Methods:**

A qualitative observational study with eleven focus groups was conducted in three locations in Southern Ecuador; a total of 110 participants the Shuar, Kichwa and Mestizo ethnic groups were included. A phenomenological and hermeneutic analysis was conducted.

**Results:**

Fourteen main subtopics around of two predefined themes, i.e., “Health” and “Illness” were identified: 1) four bodies, 2) religiosity, 3) health as a good diet, 4) health as god’s blessing or a gift, 5) health as balance/ harmony, 6) health as community and social welfare, 7) health as potentiality or a skill, 8) health as peacefulness, 9) heath as individual will, 10) illness as an imbalance, 11) illness as bad energy, 12) illness as a bad diet, 13) illness as suffering or worry, and 14) illness from God, Nature and People illness. By analysing all the topics’ and subtopics’ narratives, a health and illness definition was developed. The principal evidence for this new framework is the presence of interculturality as a horizontal axis in health. The indigenous perspective of health and illness focus on a balance between 4 bodies: the physical, spiritual, social and mental bodies. Additionally, “good health” is obtained through of the good diet and balanced/harmony.

**Conclusion:**

Indigenous healers in Southern Ecuador have views on health and illness that differ from the Western biomedical model of care. These different views must be recognized and valued in order to build an intercultural (health) system that empowers both ancestral and modern medical knowledge and healing.

## Introduction

In all societies, different views on health, illness and medicine, and thus on preventive and therapeutic approaches coexist. The Western biomedical model of care dominates over other systems and is able to resolve many health problems. However, the domination of one model can cause difficulties in diagnosis, treatment and prevention especially in places where a variety of cultures lives together. Health models must be able to respond to the biological, cultural and social diversity of all members of a society [1, 2].

Interculturality has been described as “the equitable and respectful interrelations of political, economic, social, cultural, age, linguistic, gender and generational differences established in the space between different cultures (peoples, ethnic groups) to build a just society” [3]. Indigenous communities in Latin America use the term “interculturality” to reinforce their dignity and identity, to rebuild their relation town and to achieve mutual recognition [4]. Ecuador has a “Multicultural Constitutionalism” [4], meaning that aspects of multiculturalism and communal property are formally reconized. In addition to the recognition of indigenous laws, indigenous languages and bilingual education, a “multicultural health policy” (Plan Nacional Buen Vivir - Sumak Kawsay” [5]) has been implemented.

However, indigenous communities continue to have worse health outcomes than those of ethnicities in Ecuador and across Latin America. These differences seem to be deeply rooted in socio-historical contingencies such as isolation, gender, racism and poverty [6]. In 2010, López-Cevallos and Chi [7] demonstrated that economic status was a predictor of health care utilization. They also found, however, that indigenous ethnicity was an independent negative predictor. Other barriers were also described, including difficult communication between modern healthcare providers and indigenous patients, linguistic barriers and different perceptions of health and illness between indigenous and Western healthcare providers, were also identified [8].

The World Health Organization (WHO) defines health as “a state of complete physical, mental and social well-being and not merely the absence of disease or infirmity” [9]. Some authors have argued that the WHO definition has limitations and must be updated [10]. Recently, Charlier et al. emphasized the importance of taking into account the indigenous perspective in a new definition [11].

Ecuador is a multicultural country. This multiculturalims is one reason why traditional medicine is a pillar (support) of the health system. One principal aspect of this indigenous model is that heath and illness concepts are focused on welfare and balance/ harmony with the community in a comfortable environment. “Good health is obtained through equilibrium between material and spiritual conditions and reciprocity with the society” [12].

Therefore, this study focused on exploring a definition of health and illness from an indigenous perspective to answer the following question: How do indigenous people in the Southern Ecuador define health and illness? This work is part of a larger project that studies the aspects of intercultural healthcare in Southern Ecuadorian communities [13].

## Methods

### Ethical approval

Ethical requirements of the Research Committee of the San Francisco de Quito University were fufilled and the Ministry of Public Health approved the study (CEISH USFQ 2017-059E).

### Research design

A qualitative observational study was conducted between August and October 2016 in Southern Ecuador, in the Saraguro, Yacuambi and Cuenca (Senplades, Zones 6 and 7) regions [14]. Study participants were of different ethnicities (Table 1). We invited healers from the region of Saraguro, Yacuambi and Cuenca who worked as shaman (“Yachay” / “Uwishin” or “visionarios”), herbal-healers (“Hierbateros”), midwives (“parteras”), and bonesetters (“sobadores”). The indigenous community of southern Ecuador (Saraguros, Cholos cuencanos and Shuar) was chosen because a) this community is a center for indigenous people to meet and participate in traditional ceremonies; b) the community has a council healers (consejo de sanadores) who are in regular contact through meetings and telephone; and c) this indigenous community has some degree of power in the region.

**Table 1:** Focus group interview guide

Health and illness process: What does it mean for you to be healthy? What does it mean for you to be sick? What should people do to stay healthy? What should people do to heal themselves?	
Diagnostic and treatment resources and practices: What resources do you use to diagnose diseases? What resources are used to heal?	
Views on formal systems: What do you think about doctors? When do you send a patient to the Western physician/health system?	

Focus groups were chosen as the main data collection method because this method allows participants to interact with each other in a comfortable environment and to talk about what they think and feel [15]. The aim and objectives of the study were shared with the traditional healers’ association. An agreement between the “Universidad Técnica Particular de Loja” and three traditional healers’ associations was reached prior to the study implementation. Participants were recruited on a voluntary basis and were informed that they could withdraw at any time during the discussions. All the participants gave written informed consent.

### Recruitment and setting

Purposive sampling was used to recruit the participants. All participants were healers who were recognized by the communities. To facilitate diverse discussions on diverse levels, different types of healers were recruited. The inclusion criteria were: working as a traditional healer or partera (midwife), and having capacity in the Spanish language. However, the participants’ mother language was not Spanish, which explains the grammatical errors that can be found in the quotes.

Eleven focus groups were conducted with 110 participants aged 20–70 years. The participants identified themselves as members of the Shuar, Kichwa (Cholos Cuencanos and Saraguros) or Mestizo ethnicities, which are the ethnic groups living in the Senplades regions 6 and 7 in Southern Ecuador. The focus groups lasted between 98 and 167 min, plus an additional 30 min for initial orientation and engagement with the participants. During the focus groups, all participants actively participated.

### Data collection

The focus groups started with an initial orientation (describing the aims of the study) and engagement with the participants. Additionally, informed consent and permission to audio record the discussions were requested. Participants were ensured of the confidentiality of their contributions. Each participant received a coloured dot on his/her badge that identified him/her as a particular kind of healer. The focus groups were conducted with the help of four moderators, and four observers, who took field notes. The focus group interview guide consisted of questions focused on traditional healers’ understanding of the health and illness process, their diagnostic and treatment resources and practices and their views on the on public health policies. (Table 1). This work is part of a large project and the resources of diagnostic and treatment were not included in this article.

### Data analysis

A phenomenological method was used because the study sought to explore, describe and understand people's experiences of people concerning a phenomenon and discover the elements the common of such experience [16]. An interpretative phenomenological analysis permits detailed examination and explores personal experience and exploration of perceptions of an event.

The recorded focus group data and observational notes were transcribed verbatim and analyzed using N-Vivo software. Three researchers separately and repeatedly read and coded the data and the field notes separately and repeatedly. Codes were assigned to parts of the text and distributed into main categories to form a codebook. The codebook was shared among the researchers who collectively formulated the description of the codes, until all codes were extracted. Codes were clustered and connected under broader categories trying to identify the core variables that emerged in a new theory or explanation model [17]. Connections was sought between the experiences of the participants in relation to the research question were identified. Code saturation was achieved with the fourth focus group, the remaining focus groups were analyzed until meaning saturation was reached for all codes [18].

## Results

Eleven focus groups were conducted with 110 participants aged 20–70 years (Table 2). Participants were able to meaning-make the key terms (health and illness) in their narratives. The results of the focus group were member checked by the community healers to validate the viability of the interpretations made. The healers proposed a minor change in the definition that was formulated.

**Table 2:** Focus groups characteristics

Code	Date	Place	Ethnicity^1^	Number of participants
GF1	18/08/16	Saraguro	Kic, M	10
GF2	17/08/16	Yacuambi	Sh, Kic, M	10
GF3	18/08/16	Saraguro	Kic, M	10
GF4	18/08/16	Saraguro	Kic, M	10
GF5	17/08/16	Yacuambi	Sh, Kic, M	10
GF6	08/08/16	Saraguro	Kic, M	9
GF7	17/08/16	Yacuambi	Sh, Kic, M	10
GF8	07/08/16	Yacuambi	Sh, Kic, M	10
GF9	12/10/16	Cuenca	Kic, M	10
GF10	13/10/16	Cuenca	Kic, M	10
GF11	25/10/16	Cuenca	Kic, M	11

^1^Ethnicity: Kic: Kichwa, M: Mestizos, Sh: Shuar

We identified 14 main subtopics under the two predefined topics, i.e., “Health” and “Illness” (Table 3).

**Table 3:** Predefined topics ‘health and illness’ and their 14 subtopics

Health	
Four bodies	
Religiosity	
Health as a good diet	
Health as god’s blessing, a gift	
Health as balance, harmony	
Health as community and social welfare	
Health as potentiality, a skill	
Health as peacefulness	
Health as individual will	
Illness	
Illness as imbalance	
Illness as bad energy	
Illness as a bad diet	
Illness as suffering, worry	
Illness from God, Nature and People	

The following section describes the topics with and their subtopics. Quotes are used to enrich the results; they have been translated from Spanish into English, while trying to preserve the nuances of the discourse of the indigenous community. At the end of this section, we propose a new defition of Health and Illness based on the views that emerged.

### Health and illness process

#### Four bodies

According to Andeanbeliefs, a human being is composed of four connected bodies: the physical, emotional, spiritual and mental bodies (Q1). These four bodies remain in equilibrium through give and take. (Q2). The four bodies need to be balanced (Q3). The data reinforce the importance of all bodies being in a state of completeness that allows people to give themselves to others and to be healthy. These four bodies are in harmony not only with each other but also with everything external to the being, such as nature. Furthermore, the relationship of the body with nature is bidirectional.

*Q1: “People do not get sick only in their physical body … everything spiritual and emotional matters … ,that is, all four bodies have to have energy.”*

*Q2: “What’s the health? It is the delight of our physical body and also the psychological, the spiritual … then, once we have that power we can expand because what I receive … If I return back to that integrated part, I will receive the whole part.”*

*Q3: “We can also talk about the love to humanity, that is what it is  to be healthy [ …*] *People have a joy for a relationship with all the beings that surround us; not just men or land but with the nature itself, the stars … well, that health begins to feel this spirituality change.”*

### Health as balance or harmony and illness as bad energy

For ancestral communities, health is a complex term that includes the environment in a macro sense (the community and nature) (Q4) and in a micro sense (the individual itself). Harmony is established among all levels. It is understood that for the individual to be well, the community must also be in harmony (Q5). The balance is therefore a state of tranquility and peace in which four bodies that constitute the human being, the community, nature and God are well adjusted in a state of peaceful harmony or a dynamic tranquility (“as the days and nights flow from one to the other (Q5)”). It is therefore a balance in motion, that has “ups and downs,” but that is in a continuous flow that maintains stability . When people are balanced and things go well, people surround themselves with positive energy, and they avoid disease.

Participants referred to energy (Q6) as a flowing power; bad energy can travel from one person to another causing illness and disease. Focusing on everything positive in life is essential to attract more positive strength and to keep people well (Q7). People who curse, fight or have negative thoughts attract those sorrows, creating a bad world and ultimately contributing to their own bad health. Before birth, humans are influenced by the energy of the father and the mother (Q8), and the acts of support and sustenance to the community bring people positive energy. In contrast, fighting, stealing, gossip, etc., are seen as wrong for the community, as these behaviors charge the community with negative energy and ultimately brings diseases (Q9).

Q4: “For me being healthy means, so, a person with energy, who cares about the social community well-being [ …] If they are healthy, then, all the community has to be well.”

Q5: “Of course, a balance has to include ups and downs, it cannot be only on the top, [ …] just like day and night, like the positive and negative energy, it could have sadness and it could have happiness. That is the balance for me as the days and nights flow from one to the other.”

Q6: “Talking about energy, it has an influence on people’s state, depending on the kind of energy you have, you will get sick or not and, in that sense, we need it [a good energy] to heal people.”

Q7: “In a general way, the nature is there, it just depends on people. As long as you live you have to work with the good, because the bad exists but you have to think a lot about the good and walk to infinity. “What is sown is harvested”, as we generally say … if you don’t want … keep cursing and capturing the negative.”

Q8: “Sometimes we are born with bad energies from the belly of the mothers. For example, if the father fought with the neighbor, and then he begot me, from the mother’s womb came this bad energy.”

Q9: “Some people have bad energy, [ …] with a word wither and destroy, those are bad energies. Then, some illness comes from hate, revenge, envies, fights, naggings, slanders, gossip …”.

### Health as peacefulness

In on sense, an essential aspect of health is to be calm within oneself, in the family and the community and to share and not to be selfish. Peace is an indispensable element of well-being (Q10). The healing capacity of healers is associated with the ability to convey tranquillity, love and patience to the patients (Q11). Restlessness, worry, having repetitive thoughts about problems is a severe psychological illness.

Q10: “To be healthy is to be at peace with yourself, with the nature and with others.”

Q11: “You have to be willing, supportive, quiet … to have love and patience with the patients.”

### Health as “individual will”

The strength of the individual is related to health; as long as people consider themselves to be healthy and strong, this well-being will remain (Q12). Thus, being healthy also depends on the decision of each person and volition (Q13). Healers convince patients to say to themselves, “I can” as a form of self-empowerment (Q14). Self-esteem and conviction in the potential of oneself are also important for the effectiveness of therapeutic remedies (Q15).

Q12: “If you say “I’m going to go wrong”, automatically you are bad, if someone has the flu, […] and I say “He already contaminated me” so I will be contaminated because that person is taking part of the discomfort by saying that is getting worse, but if I say “That’s his problem, I’m ok” I’ll be fine. The psychological issues influence a lot”.

Q13: “I believe nothing is going to happen to me in life, that I’m going to live happily, [ …] as the proverb says, “who want to suffer suffers, who want to enjoy enjoys”.

Q14: “We explain to the person to say: “I can”, No one can act against you because you stay strong. Weak people fall again into any mistakes they make and the person sinks.

Q15: “We should have affection for ourselves, self-esteem must be high. [ …] Then, if you have healthy spirit and mind, food will do its part and everything will make you feel good.”

### Health as a good diet and illness as a bad diet

Food is tremendously important to the indigenous community. A good allows a person to maintain health and balance. Food includes ingestion as well as cultivation and harvesting processes, always in relation to the “Pachamama” (Earth Mother). Participants described their “own food products” that are natural and without chemicals (Q16). They use traditional utensils such as clay pots, stone cookware and leaves (Q17). Throughout the whole process of production, including the cultivation, planting, breeding, harvesting and preparation of food, good and bad energy is transmitted (Q18). Therefore, people in the indegenous community place importance on blessing and thanking the food and assert that one should not cook or to eat with bad energy or annoyances (Q19).

Modernity is associated with a poor diet. The new generations, instead of eating the natural products, they eat “lollipops, yogurts” or “eat only rice”. Participants also discussed the poor quality of food that they have to buy in markets when they finish their own harvested food was also discussed. The lack of a diverse diet, without vegetables and grains from the earth, as well as eating with bad energy or without thankfulness is associated to the appearance of diseases and with a state of weakness of the person.

Likewise, all types of traditional healers give advice related to food to their communities; healers recommend following a good diet to stay strong (Q20) and to prevent diseases, and they suggest specific foods for to particular ailments or diseases.

Q16: “We don’t use chemicals. We plant and use organic fertilizer and that’s why we are a bit stronger, [ …] our fathers raised us with our own grains. We have sown corn, “poroto”, “oca”, potato, “melloco”, “mashua” … everything from here.”

Q17: “The ancients lived longer because they cooked in a clay pots, stone cookware and leaves. Our medicine is slow but it is safe.”

Q18: “When you buy a bottle of water it is dead water, although they say it’s virgin water or whatever, that water is dead by the time you put it in a bottle because it loses all the nutrients and the spirit. [ …] You should drink putting the sense, the energy.”

Q19: “That’s why we have good manners and we invoke the creator [ …] to bless the food and take away the bad energy. It’s the same with medicines.”

Q20: “When you give medicines you have to support it a lot with meals, [ …] Madam, if you want to get better you have to stop eating that food.”

### Health as God’s blessing or a gift

Health is a blessing or a divine gift, which is given to humans to be able to surrender to others. Health also depends on a bidirectional connection with the environment (Q21–22). People are healthy thanks to the gift of Earth Mother (Q23), Pachamama, who offers us everything that we need to feel fulfilled and in harmony. God is the giver of life and harmony. In addition, many healers consider that their ability to heal comes directly from God and that it has been bestowed upon them, while others focus more on “the Gift” as ancestral and generated through community learning (Q24). Participants spoke of God as a protector, who does not only grants health but also attributes the ability to maintain health and to prevent disease. Illness is accepted when it is understood that God has sent it for some reason (Q25). Belief and faith are important because they keep people strong to deal with bad energy and thus avoid disease (Q26).

Q21: “We thank God because we are improving our patients’ care, encouraging them through alimentation. For example, during labor, we use our typical meals … but in the hospital … it’s not the same, they give you whatever food and the patient will be weaker.”

Q22 “Health is the environment where we delight all the elements given by our Nature Mother.

Q23: “I pray in the name of God, son and the holy spirit. Father help us, my God, oh almighty, you put all things and stars soul and spirit … please lead us, deliver us from all the evil spirits.”

Q24: “God has given each of us a Gift, then, the people go to the wise, healers, “sobadores”, then, we go there because they can heal us thanks to God.

Q25: “God forgive me, but I don’t believe in evil … if God allows bad things happens to me it is because he wants it.  Then I have a strong faith no one can make me nothing, just God.”

Q26 “To stay healthy you must believe in God and everything is going to be ok. They can destroy me but if I firmly believe in God, that he has created me, that he gives me strength, he has given to me the intelligence. Then, I am not so sensitive to taking that bad energy, I must first protect myself with my prayers when I wake up and go to bed.”

Health is associated with productive capacity and conflict resolution (Q27), as well as the ability to face problems and being able to overcome them (Q28). Disease deprives people of the pleasure and fulfilment that is felt when they do useful things, and thus is associated with the subjective perception of incapacity. It is important to be able to contribute to others through productive activity, and work. Being healthy individually is critical mainly because of the repercussions it has for the community.

Q27: “Well, for me being healthy is also being able to confront everyday problems, to face the upcoming problems, to know … how to face it. Do not truncate us to that.”

Q28: “Health is the fundamental base that we feel right, that we are working”.

The origin of diseases is also seen as a multidimensional factor. A person also has the capacity to destroy and produce disease. In addition, certain diseases arise from interaction with nature or are established by God. Thus, a person’s ability to heal certain diseases is born. The disease of humankind is determined by envy, witchcraft, and the transmission of bad energy (Q29).

Q29: “Through spells we heal first the disease which has been done by humans’ evil, not a nature disease, not from God, but done by witches.”

## Discussion

In this study, we aimed to develop a more comprehensive definition of health and illness from the perspective of indigenous people in southern Ecuador. Our qualitative research is important to understand the concepts of health and illness in the Ecuadorian indigenous sociocultural and linguistic context. Although an understanding of the differences in health concepts determines the success of an intercultural model, research into indigenous health has been largely focused on non-indigenous, rather than on indigenous notions of health [19]. In a descriptive study about the facilitators of and barriers to the implementation of intercultural health policy in Chile [20], one of the barriers identified was the hegemony of western  medicine and an incapacity to understand and include the conceptualization of health and illness according to the indigenous cosmovision.

At the 51st World Health Assembly [21], member States were urged “to develop and implement, in close cooperation with indigenous people, national plans of action or programmes on indigenous people’s health which focus on ensuring access of indigenous people to health care”. In Ecuador, since the approval of the Ecuadorian Constitution in 2008, public policies have aimed to establish the “Sumak Kawsay” or “Good-living”, an alternative development view that considers a multinational and intercultural approach [22]. To do so, the government created the MAIS-FCI (Family, Community and Intercultural Model for an Integral Care) in 2012. This model provides an intercultural approach as “an ethical and political position of recognition and respect for diversity that allows a horizontal and synergistic interaction”. Moreover, this system is grounded in the recognition of the fact that the modern biomedical approach is unable to fully “understand, respect and incorporate the ancestral knowledge and practice” necessary to facilitate health care access to all Ecuadorians [23]. By understanding the subtopics of our research, we can define the two main concepts, health and illness.

### Health definition

Health is an environment of mental, emotional, physical and spiritual balance that leads to a life with positive energy, peacefulness and harmony with the community, nature and oneself and is achieved through good nutrition, sharing, self-will and faith. It is a blessing that empowers us to work for social welfare and constitutes one of the fundamental bases for indigenous people and all who live on our planet Earth.

### Illness definition

Illness is the imbalance of the four bodies (mental, emotional, physical and spiritual) that comes from God, from nature or from abuses among human beings and causes malaise, decay, selfishness and an inability to do things for oneself and for others. This loss of physical and psychological well-being causes a chaotic situation of weakness, discomfort and low self-esteem that makes it impossible to build and respond to the community, causing the destruction of oneself, others and the entire Pachamama.

Our results of the analysis of health-illness concepts from the healer’s perspectives agree with the 1999 Report of the International Consultation on the Health of Indigenous Peoples, which defines health “in holistic terms and emphasizes the interconnectedness of physical, spiritual, mental and emotional health” [24]. In addition, this report highlights the cultural, political and social dimensions of indigenous communities and the importance of these communities controlling their own development land and natural resources, further evidencing that the health of the individual is dependent on the overall health of his or her community. Despite of the time that has passed since the publication of this report, there has been little movement by the WHO to incorporate these different worldviews. Recently, in their open letter to the WHO, Charlier et al. (2017) called for the incorporation of indigenous perspectives to enrich a future definition of health. They included environmental equilibrium, spirituality and adaptation in their definition: “healthy is one who is balanced on his own, with others and the environment, but also able to adapt to things” (11). The authors of the present paper support this statement and argue for a definition of health that includes all people through participatory qualitative research processes.

In the Kichwa language, there is no translation of the word “health”. The indigenous people in the Andes understand health as a holistic “well-being next to the whole” [7]. Families and communities play a central role, and balance and equilibrium with everything external to the being and within one's inner world are important. These characteristics are consistent with our results, and reinforce this holistic perspective.

Unlike in the hegemonic Western thought, in which reality is symbolized in dichotomous dualities, in the indigenous cosmovision, health and illness are not just opposites but parts of the same continuum. This conceptualization makes it easier to assume a complementary cooperation between traditional medicine and biomedicine.

The current thinking of indigenous people in Southern Ecuador is consistent with the concepts of the classical Kichwa cosmovison, but our study also revealed some specific issues. The most obvious difference is the incorporation of God in the narratives. The Christian religion is sometimes mixed with pre-Hispanic symbols and Catholic prayers have been included alongside ancestral rites in the healing process. Both seem to coexist harmoniously . The importance of food in the health-illness process is another issue that arose in this study. In Orr’s [25] publication about “Food, Healing, and Hunger in Quechua Narratives of Madness,” three overlapping categories emerged: food as symptoms, as an explanatory model, and as a facet of healing. In our study, we observed that nutrition is also referred to in these three ways; participants thought that poor nutrition creates illness, that a sick person cannot “eat with pleasure” and that food is also used as an important part of the healing process. Eating is a key element in the well-being narratives for indigenous people, and it needs to be included in order to provide culturally appropriate care.

Our study has some limitations. First, it only considers the narratives of the indigenous people of Southern Ecuador in two Senplades regions (6–7). Furthermore, the differences between the cosmovisions of the Shuar, Kichwa and Mestizo nationalities were not investigated. Another limitation is that only Spanish speaking traditional healers were recruited, and therefore some other more traditional healers were not included.

On the other hand, we consider that the definitions were formulated in collaboration with all parties (rural doctors and traditional healers), and not just by using healers as study objects. Further, the results were member checked by the healer communities of Saraguro to validate the viability of the interpretations made. The community proposed only a minor change in the definitions that were incorporated. The diversity between researchers is also important for triangulation; they come from three different universities and three different countries, and some have worked with indigenous communities prior to this project.

We hope that the results of this study are a step towards and a contribution to the development of a new WHO definition of health that incorporates the concepts and views of indigenous people. On a local level, this article is a starting point for intercultural collaboration between healers and physicians in our region. It will facilitate appropriate communication, mutual respect and interaction in a model in which healers and physicians make a joint effort to improve local health care.

## Conclusions

Developing inclusive health plans that take into account all the people’s perspectives and that have respect for each culture is critically important for health. Qualitative research can play a key role in including indigenous people as active collaborators in the construction of their own perspective of health and illness based on their own linguistic and sociocultural references. For the indigenous people of Southern Ecuador, health and illness have different meanings that must be valued to build an intercultural system that empowers ancestral knowledge and indigenous people.

This paper contributes to the construction of definitions of health and illness from a Southern Ecuadorian indigenous perspective, and ca be an essential step in collecting the perspectives of different cultures to avoid the imposition of Western cultural concepts and constructs of health.

## Abbreviations

Kic: Kichwa
M: Mestizos
MAIS FCL: Family, Community and Intercultural Model for a Integral Care
Sh: Shuar
WHO: World Health Organization
